# 

**Published:** 1888-02

**Authors:** 


					﻿Entered at the Post Office at Austin. Toras, as Second Class Man Matter.
Vol. III
FEBRUARY., 1888.
No. 8.
DANIEL'S
MEDECAL
JOURNAL
F. E. DANIEL., M.D., Editor and Publisher, AUSTIN, TEXAS.
Northern Office, 1217 Filbert Street, Philadelphia.
Eugene Von Boeckmann. Steam Book and Job Printer. Aurtin, Texas.
				

